# Diagnostic Imaging of Extrapulmonary Tuberculosis Across Organ Systems

**DOI:** 10.3390/diagnostics16040586

**Published:** 2026-02-15

**Authors:** Madeleine T. Dang, Kara Lukas, Daniel H. Choi, Timothy J. Chu, Vishwanath Venketaraman

**Affiliations:** College of Osteopathic Medicine of the Pacific, Western University of Health Sciences, Pomona, CA 91766, USA; madeleine.dang@westernu.edu (M.T.D.); kara.lukas@westernu.edu (K.L.); daniel.choi@westernu.edu (D.H.C.); timothy.chu@westernu.edu (T.J.C.)

**Keywords:** extrapulmonary tuberculosis, diagnostic imaging, magnetic resonance imaging, computed tomography, ultrasound, meningeal tuberculosis, tuberculous lymphadenitis, abdominal tuberculosis, genitourinary tuberculosis

## Abstract

Extrapulmonary tuberculosis (EPTB) is an infectious disease characterized by the invasion of *Mycobacterium tuberculosis* beyond the lungs. Diagnosis is frequently delayed due to nonspecific clinical presentations that vary by organ system, making diagnostic imaging essential for disease detection, characterization, and treatment monitoring. The objective of this review is to examine and summarize imaging-based approaches for the diagnostic evaluation of EPTB across multiple body systems, including the central nervous system, spine, cardiovascular system, lymphatic system, abdominal and hepatic organs, genitourinary tract, cutaneous and soft tissue, and other rare sites. While computed tomography, magnetic resonance imaging, positron emission tomography, and ultrasound are widely used in the evaluation of EPTB, their ability to provide a definitive diagnosis is often limited by nonspecific radiologic findings. Emerging techniques, including perfusion-weighted MRI, contrast-enhanced ultrasound, and machine learning, have been discussed, as they improve lesion characterization and EPTB differentiation. By organizing imaging findings according to affected organ systems, this review highlights both shared diagnostic challenges and site-specific patterns that can inform clinical suspicion. Together, these developments underscore the value of a multimodal, organ-specific imaging approach integrated with the clinical context to improve the recognition and management of EPTB.

## 1. Introduction

Tuberculosis (TB) is a major, ongoing global health challenge and continues to cause significant morbidity and mortality worldwide despite advances in diagnosis and treatment [1,2]. TB is caused by *Mycobacterium tuberculosis*, a slow-growing, acid-fast bacillus that most commonly affects the lungs. The disease may disseminate beyond the pulmonary system resulting in extrapulmonary tuberculosis (EPTB) [2]. EPTB accounts for approximately 15–20% of all cases of TB and can occur during primary infection, reinfection, or reactivation of latent infection [1].

EPTB is frequently underdiagnosed or subject to delayed diagnosis, typically discovered following the development of complications [1]. Diagnostic delays arise due to nonspecific, overlapping presentation with a broad range of infectious, inflammatory, and malignant conditions, as well as a low bacillary load and involvement in sites where sampling is difficult [3,4]. In a recent prospective study, Sinha et al. [3] reported a median diagnostic delay of four weeks, with most cases relying primarily on clinical and radiological evaluation rather than microbial confirmation [3].

EPTB diagnosis relies on a composite assessment of clinical, laboratory and histological findings, rather than readily available microbiological confirmation as in pulmonary TB. Therefore, imaging facilitates accurate anatomical localization of EPTB, supports earlier and more precise diagnosis, guides tissue sampling, and aids in treatment planning and monitoring [1]. Conventional imaging modalities such as computed tomography (CT), magnetic resonance imaging (MRI), and ultrasound remain central in the diagnosis of EPTB; newer approaches including positron emission tomography/computed tomography (PET/CT), contrast-enhanced ultrasound (CEUS) and diffusion tensor imaging (DTI) have shown potential in improving early, non-invasive detection and characterization of disease [5,6,7,8,9].

The objective of this review is to explore, summarize, and evaluate recent advances and current findings in diagnostic imaging of EPTB, including both conventional and advanced modalities, while examining their clinical utility in early detection, anatomical localization, and characterization of imaging features across different organ systems.

## 2. Methods

We conducted a literature search for this narrative review in PubMed, Cochran, and Embase from September 2025 through 3 February 2026, using the terms “diagnostic imaging for extrapulmonary tuberculosis” as well as the terms “imaging” and “radiology” in conjunction with “extrapulmonary tuberculosis”, “meningeal tuberculosis”, “cutaneous tuberculosis”, “tuberculosis lymphadenitis”, “abdominal tuberculosis”, “tuberculous pericarditis”, and “genitourinary tuberculosis”.

Articles were screened based on title and abstract, followed by full-text review. We included studies primarily published within the last five years that were relevant to diagnostic imaging of EPTB. We excluded studies that were not related to imaging of EPTB or that only discussed pulmonary TB.

## 3. General EPTB Imaging

EPTB can affect nearly any organ in the body, with imaging playing a central role in diagnosis, treatment planning, and monitoring [10]. Specifically, MRI and CT offer detailed evaluation of organ involvement while ultrasound provides a rapid, accessible screening tool, particularly for superficial lesions and point-of-care assessment [11]. Because imaging findings are often non-specific and can vary by organ, awareness of organ-specific patterns is important to improve detection [12].

Ultrasound, including point-of-care ultrasound (POCUS), helps with the diagnosis of EPTB through the identification of common but often non-specific features, including lymphadenopathy, pleural effusion, and organ lesions [13]. A retrospective analysis by Nacarapa et al. [14] found that POCUS is most effective in detecting abdominal TB and screening for pleural TB and pericardial TB; however, it offers limited diagnostic benefit for other forms of EPTB [14]. POCUS protocols, such as focused assessment with sonography for HIV and Tuberculosis (FASH) and extended FASH (eFASH), improve detection in high-risk populations and facilitate guided biopsies, especially when combined with CEUS to distinguish necrotic tissue [13]. While ultrasound alone cannot confirm EPTB, it is a valuable, accessible screening and monitoring tool [13]. Evidence from a 2023 randomized controlled, parallel, superiority trial demonstrated that while eFASH did not show superiority in the correct management of EPTB, the absence of complications after ultrasound-guided invasive procedures suggests that ultrasound guidance can help increase the proportion of patients with definitive TB [15].

In contrast to ultrasound-based approaches, molecular imaging with PET/CT has emerged as a promising adjunct for assessing disease burden and treatment monitoring in EPTB. Dynamic positron emission tomography (PET) imaging using radiolabeled anti-tuberculous agents has demonstrated the ability to quantify compartment-specific drug distribution in vivo, revealing marked heterogeneity in antibiotic penetration across extrapulmonary sites, including the central nervous system (CNS), highlighting its potential role in optimizing therapeutic regimens for complex forms of TB [16].

More specifically, studies evaluating ^18^F-fluorodeoxyglucose (^18^F-FDG) PET/CT have demonstrated its ability to map whole-body disease burden and identify a greater extent of extrapulmonary and multi-organ disease than is suspected on clinical assessment alone. Additionally, ^18^F-FDG PET/CT can track longitudinal metabolic changes during therapy [17,18]. Despite promising advances in PET/CT imaging for EPTB, several limitations remain. A major challenge is the lack of specificity of ^18^F-FDG PET/CT. For instance, one case report demonstrated that disseminated TB can mimic metastatic cancer on PET/CT, emphasizing that imaging findings alone may be misleading and require histopathological confirmation [19].

From a safety perspective, cumulative radiation exposure remains a concern during longitudinal imaging for treatment monitoring, particularly with repeated CT or PET/CT examinations [1]. MRI offers high spatial resolution without ionizing radiation, making it an attractive alternative for follow-up imaging when available [5]. Continued research into radiation-sparing imaging strategies for treatment monitoring is therefore warranted.

Emerging artificial intelligence (AI) frameworks integrating radiomics, deep learning, and clinical data have recently shown accuracy in differentiating spinal TB from pyogenic spondylitis using conventional chest X-rays (CXR). These multi-modal AI models can detect subtle imaging patterns beyond human perception, improve diagnostic confidence, and potentially reduce the need for invasive tissue sampling, thereby offering a complementary approach to molecular imaging in the non-invasive assessment of EPTB [20]. Despite these advantages, AI models are not free from bias, as their performance depends on the quality and diversity of the training data. Skilled interpretation remains essential to contextualize AI outputs, validate findings, and integrate imaging results with clinical and laboratory information in order to ensure accurate and reliable diagnosis [21,22].

## 4. Central Nervous System Tuberculosis

CNS TB is a serious and potentially life-threatening form of EPTB, resulting from dissemination of *M. tuberculosis* from a primary infection site to the brain or spinal cord [5]. CNS TB can manifest in several forms, including meningeal, parenchymal, pituitary, and spinal involvement [5,23,24,25]. Early detection of CNS TB can be challenging because cerebrospinal fluid (CSF) analysis may only reveal trace amounts of *M. tuberculosis* DNA. Consequently, imaging modalities are routinely used in the evaluation of all cases of CNS TB [3,25].

### 4.1. Conventional Magnetic Resonance Imaging

#### 4.1.1. Cranial Meningitis

**Figure 1:**
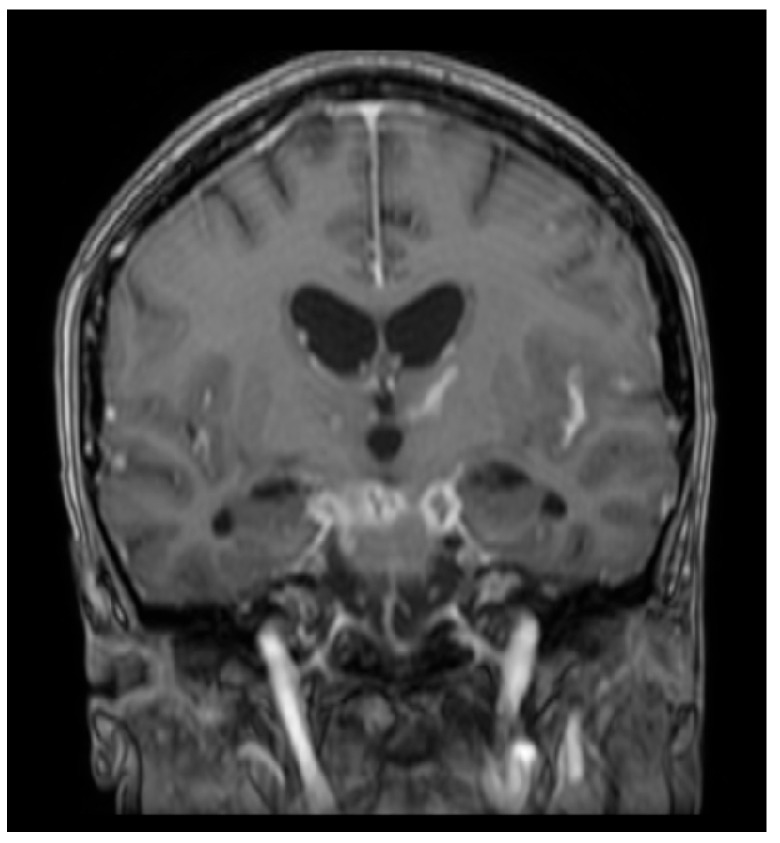
Post-contrast T1-weighted MRI of a patient with tuberculous meningitis demonstrating marked leptomeningeal enhancements and enhancing basal exudates. Case courtesy of Ammar Haouimi, Radiopaedia.org, rID: 72617, licensed under CC-NC-BY-SA 3.0 [27].

TB meningitis, the most common subtype of CNS TB, occurs due to the rupture of meningeal or parameningeal granulomas in the subarachnoid space and presents with symptoms including fever, headache, vomiting, and focal neurological deficits [1,3,5]. A 2020 double-blind clinical trial evaluated MRI brain findings of TB meningitis at baseline and after two months of anti-TB treatment. The most common baseline findings were tuberculomas and meningeal enhancement, primarily in the basal meninges and sylvian fissure. However, many patients developed paradoxical reactions after two months of treatment with radiological findings of new tuberculomas and meningeal enhancement [5]. Similarly, a 2021 prospective study reported that the most common MRI findings were leptomeningeal enhancement and tuberculoma (Figure 1) [26]. Patel et al. [23] further characterized these findings and found that the most reliable MRI findings in tubercular leptomeningitis involving the pia mater and arachnoid mater were diffuse enhancements of exudates in the basal cisterns and diffuse leptomeningeal enhancements in the affected area (Figure 1). Additionally, the study found that in tubercular pachymeningitis involving the dura mater, common MRI findings were post-contrast dural enhancements and focal or widespread dural thickening [23]. MRI evaluation after two months of treatment showed that 89% of patients had new or worsening MRI findings while only 39% had worsening clinical symptoms; most had new or enlarged tuberculomas and new or thicker meningeal enhancement [5].

#### 4.1.2. Intracranial Parenchyma

Parenchymal TB findings vary depending on the stage of the lesion. In the caseating phase, T2-weighted images show hypointensity, which reflects fibrosis, sclerosis, and free radical activity from macrophages. The surrounding edema appears hyperintense during this phase but tends to resolve as the lesion progresses to the calcified stage [23].

#### 4.1.3. Pituitary Gland

In 80–90% of healthy children and adults, the posterior pituitary appears hyperintense on T1-weighted mid-sagittal MRI images, a feature known as the posterior pituitary bright spot (PPBS). A 2022 retrospective, case–control study analyzed brain MRIs from patients with CNS TB and controls with normal MRIs to determine the odds of absent PPBS. The study found that the odds of lacking a PPBS in CNS TB was 7.90. Furthermore, incorporating the absence of a PPBS as a radiographic diagnostic feature increased the diagnostic yield from 77% to 84%, with a specificity of 91.5% [24].

#### 4.1.4. Spinal Meningitis

Tubercular spinal meningitis involves infection and inflammation of the spinal meninges and is often accompanied by symptoms such as headache, vomiting, altered consciousness, and lumbar back pain [25]. MRI findings include linear dural enhancement with occasional leptomeningeal nodular enhancement and spinal edema [25]. In a 2020 comparative study, intraspinal TB was characterized by thickened meninges and nodular, poorly defined extrapulmonary lesions; these findings help differentiate it from intraspinal metastatic disease, which has a greater number of intramedullary lesions with sharp margins [28]. The authors also found that intraspinal TB lesions have more obvious enhancement and thicker meninges compared to intraspinal metastatic lesions [28].

#### 4.1.5. Spinal Parenchyma

TB infection of the spinal cord parenchyma, or tuberculous myelitis, is considered a less common manifestation of EPTB. It is characterized by inflammation of the spinal cord and often occurs alongside meningeal disease [29]. MRI commonly shows linear dural enhancement, CSF loculations, arachnoiditis, and tuberculomas [29]. Parenchymal lesions are typically located in the cervical or thoracic spinal cord, appearing isointense or slightly hyperintense on T1-weighted images and isointense to hypointense on T2-weighted images. Caseating tuberculomas show T1 isointensity to hypointensity and a T2 hypointense core with a hyperintense rim [25]. Longitudinal extensive transverse myelitis (LETM), defined as inflammation of the spinal cord involving three or more contiguous vertebral segments, is a common finding and most often involves the cervicodorsal spinal cord [29]. In regions where TB is endemic, TB should be included in the differential diagnosis of LETM, as its imaging appearance can mimic other demyelinating or inflammatory myelopathies [29].

### 4.2. Diffusion Tensor Imaging

DTI is an advanced MRI technique that assesses the Brownian motion and directional movement of water molecules, which allows detection of early changes in white matter tracts, even before they become apparent on conventional MRI [30]. A 2024 cross-sectional, observational study reported a significant reduction in FA values, a measure of microstructural integrity, across the majority of white matter tracts in patients with CNS TB compared to healthy controls; these reductions were correlated with increasing clinical severity of TB meningitis [30]. Key imaging findings for CNS TB across different imaging modalities, including DTI, are summarized in Table 1. Additionally, Wang et al. [6], employing tract-based spatial statistics, found that the most prominent white matter changes were located in the corpus callosum and corona radiata, suggesting that these microstructural changes may explain the cognitive dysfunction observed in patients with intracranial TB [6]. Together, these studies highlight the potential of DTI to detect subtle white matter injury and its value as a biomarker for disease severity and neurocognitive outcomes in CNS TB.

### 4.3. Positron Emission Tomography/Computed Tomography

A 2021 prospective study evaluated the ^18^F-FDG PET/CT in patients with TB meningitis. Both whole-body PET/CT and brain MRI were performed on all patients. While all patients demonstrated abnormal MRI findings, PET/CT was abnormal in 92% of cases and showed greater sensitivity for detecting extracranial involvement, particularly in the lymph nodes, compared to intracranial lesions. These results suggest that PET/CT may serve as a sensitive tool for identifying extracranial TB involvement [26].

### 4.4. Advanced Imaging and Prognostic Modeling

Recent advances in neuroimaging analysis have focused on improving the diagnostic accuracy and prognostic assessment of CNS TB [31,32]. Canas et al. [31] proposed a new prognostic model for the diagnosis of TB meningitis that integrates longitudinal imaging data with clinical parameters, demonstrating that imaging features such as basal ganglia hyperintensities, hydrocephalus, and tuberculoma significantly improve diagnostic precision [31]. Similarly, Ma et al. [32] introduced a fully automatic, deep learning-based radiomics pipeline capable of identifying changes in the basal cistern that are imperceptible to the naked eye [32]. These approaches highlight the potential of advanced imaging analytics to refine diagnosis and support the prediction of clinical outcomes in patients with CNS TB [31,32].

Future research should continue to explore the integration of advanced MRI techniques, such as DTI, with PET/CT to improve early detection and treatment monitoring of CNS TB.

## 5. Spinal Tuberculosis

TB infection of the spinal vertebrae, or tuberculous spondylitis, often presents with back pain, progressive neurological deficits, and spinal deformities [33]. Many spinal diseases can mimic TB and must be ruled out before initiating anti-tubercular treatment. A combination of imaging and tissue biopsy provides sufficient information to avoid the extensive testing otherwise required to exclude other spinal diseases [33,34].

### 5.1. Conventional Magnetic Resonance Imaging

**Figure 2:**
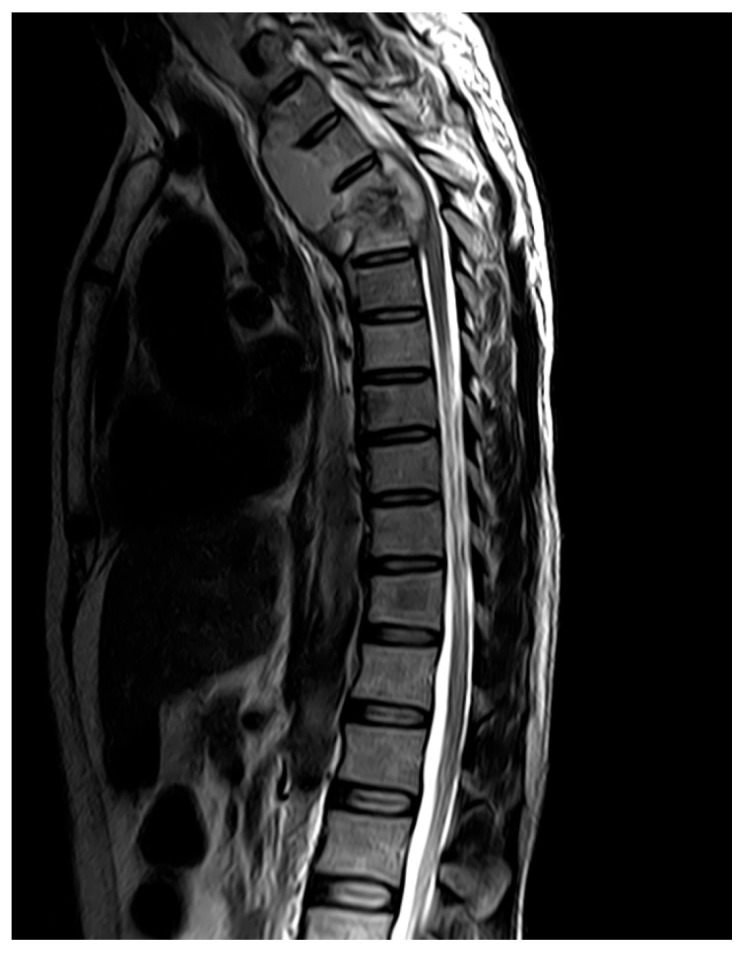
T2-weighted MRI of the thoracic spine in a patient with tuberculous spondylitis showing destruction of the T4 and T5 vertebral bodies and associated prevertebral and paravertebral abscess formation. Case courtesy of Pir Abdul Ahad Aziz Qureshi, Radiopaedia.org, rID: 73438, licensed under CC-NC-BY-SA 3.0 [38].

In a retrospective study of 80 patients, MRI identified bony involvement and associated pre- and paravertebral complications, with recommendations to perform whole-spine screening using sagittal post-contrast T1-weighted fat-saturated sequences to detect subtle lesions [35]. Tuberculous spondylitis can be differentiated from pyogenic spondylitis based on imaging features, as it more frequently involves multiple vertebral bodies in the thoracic spine or at the T12/L1 junction, exhibits skip lesions and causes severe vertebral destruction [36]. Additionally, paravertebral abscesses in tuberculous spondylitis present with clear boundaries, thin and smooth walls, and contrast enhancement of the anterior longitudinal ligament (Figure 2) [36]. These imaging findings are summarized in Table 2. MRI demonstrates excellent sensitivity (96–100%) and specificity (88–93%) in tuberculous spondylitis [33,37].

### 5.2. Perfusion-Weighted Magnetic Resonance Imaging

Perfusion-weighted MRI tracks the flow of a contrast agent through blood vessels and measures microcirculatory blood flow [39]. A 2022 study in a rabbit model of spinal TB found that parameters including Efirst, Ee, PH, MSI, Emax and SER, may have potential as early diagnostic markers for spinal TB [39]. Although this approach is currently experimental, it shows promise for early diagnosis before structural damage becomes evident [39].

Future studies should evaluate advanced MRI techniques in humans, including the translation of experimental perfusion biomarkers into clinical practice, to assess their use in early detection of tuberculous spondylitis.

## 6. Tuberculous Pericarditis

Cardiac involvement in TB most commonly manifests as tuberculous pericarditis, with imaging playing a key role in diagnosis, disease management, and longitudinal assessment [40].

### 6.1. Transthoracic Echocardiography

Transthoracic echocardiography (TTE) remains the first-line modality due to its accessibility and ability to characterize pericardial effusion and constrictive physiology [40,41]. In a retrospective cohort study of immunocompetent patients with tuberculous pericarditis, serial echocardiography demonstrated that constrictive features often improve with appropriate anti-tuberculous therapy, with over 80% of patients showing resolution by six months of treatment [42]. Importantly, individuals presenting with isolated pericardial effusion rarely progressed to chronic constrictive pericarditis when treated with anti-TB drugs, further emphasizing the prognostic value of early TTE imaging in guiding treatment and prevention of complications such as constrictive pericarditis [42].

### 6.2. CT, ^18^F-FDG PET, and MRI

**Figure 3:**
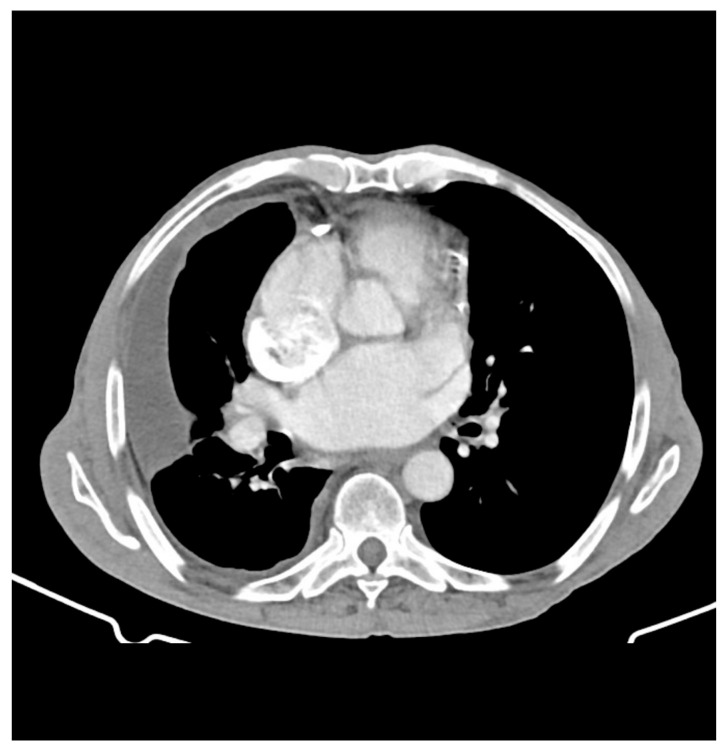
Axial CT view of a patient with likely TB showing pericardial thickening and scattered calcifications, which are characteristic findings of tuberculous pericarditis. Case courtesy of Michael P. Hartung, Radiopaedia.org, rID: 72955, licensed under CC-NC-BY-SA 3.0 [44].

In addition to echocardiography, cross-sectional imaging techniques such as cardiac CT, ^18^F-FDG PET, and cardiac MRI provide complementary insights into anatomy and tissue characteristics [40,43]. A study by Du et al. [43] showed the utility of CT in detecting pericardial thickening, calcification, and associated mediastinal lymphadenopathy, features that support a tuberculous etiology in endemic regions (Figure 3). The same study also assessed ^18^F-FDG PET imaging, which revealed diffuse pericardial uptake of ^18^F-FDG in 77.8% of patients with confirmed tuberculous pericarditis [43]. Combining ^18^F-FDG PET and CT yielded a high diagnostic sensitivity of 82% in a cohort of 11 patients, illustrating how the integration of both metabolic and anatomic imaging can improve diagnostic confidence [43]. Furthermore, cardiac MRI has been described as useful for evaluating the extent of pericardial involvement, particularly in cases of constrictive pericarditis or when suspicion of myocarditis is high [40]. Overall, these imaging modalities help facilitate earlier diagnosis of tuberculous pericarditis and guide therapeutic management. Key findings across various imaging modalities used in the diagnosis of tuberculous pericarditis are summarized in Table 3.

## 7. Tuberculous Lymphadenitis

Tuberculous lymphadenitis is the most common manifestation of EPTB, with potential complications ranging from rupture to the formation of sinus tracts [1]. Despite its prevalence, existing diagnostic techniques to distinguish tuberculous lymphadenitis from other causes of lymphadenopathy lack specific imaging signatures and often rely on invasive procedures [45,46,47]. These limitations highlight the need for more effective and standardized imaging techniques to support clinical management. Key imaging findings across ultrasound and CT for tuberculous lymphadenitis are summarized in Table 4.

### 7.1. Ultrasound

Efforts to improve diagnostic imaging of tuberculous lymphadenitis have focused on identifying ultrasound-based features that may enhance disease assessment. A retrospective study by Zhao et al. [45] identified specific multimodal ultrasound features associated with cervical tuberculous lymphadenitis rupture, such as unclear margins and heterogeneous internal echotexture, suggesting that sonographic markers may aid in predicting disease complications. This multimodal prediction of lymph node rupture displayed a sensitivity of 89.29% and specificity of 100% [45]. Similarly, Yu et al. [47] evaluated multimodal ultrasound parameters in 72 patients and found that changes in intranodal pus, surrounding tissue echogenicity, and elasticity scores differed significantly between treatment-responsive and nonresponsive cases, highlighting ultrasound’s potential role in monitoring therapeutic response.

**Figure 4:**
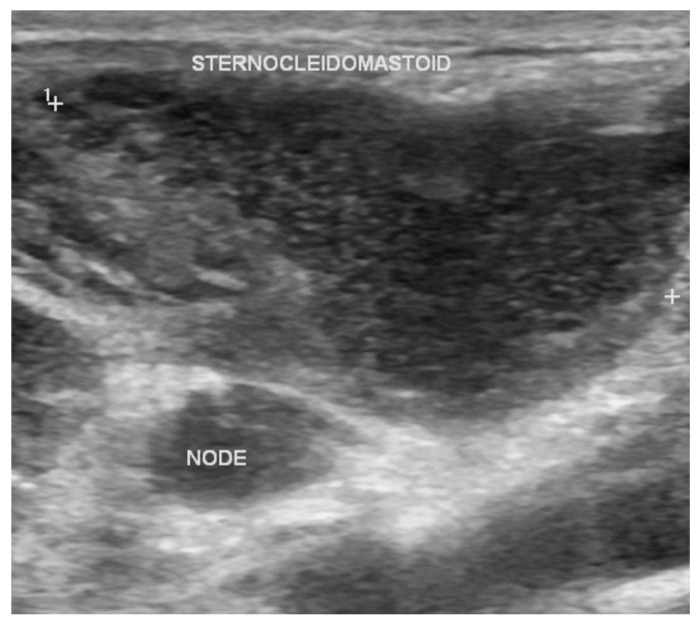
Ultrasound image of the neck demonstrating an enlarged hypoechoic cervical lymph node adjacent to the sternocleidomastoid muscle, consistent with cervical tuberculous lymphadenitis. Case courtesy of Maulik S Patel, Radiopaedia.org, rID: 19137, licensed under CC-NC-BY-SA 3.0 [50].

Beyond conventional ultrasound techniques, functional and contrast-enhanced techniques have aided in expanding the scope of imaging capabilities. Shear wave elastography has shown promise as a potential radiologic marker for predicting the therapeutic response of tuberculous lymphadenitis [48]. Additionally, CEUS have shown that features such as peripheral rim-like enhancement and internal heterogeneous enhancement occur more frequently in cervical tuberculous lymphadenitis than in non-tuberculous lymphadenopathy, supporting CEUS as an adjunct for diagnostic evaluation; combination of both features yielded a diagnostic sensitivity of 74.23% and specificity of 94.34% (Figure 4) [49].

### 7.2. CT, ^18^F-FDG PET

CT imaging has also been shown to provide insight into disease behavior. For example, one study revealed that abdominal tuberculous lymphadenopathy exhibits distinct distribution patterns depending on hematogenous versus nonhematogenous dissemination, suggesting that CT may help infer underlying mechanisms of spread [9].

Despite advances in functional imaging, ^18^F-FDG PET/CT remains limited by poor specificity in extrapulmonary manifestations of TB, including lymph node TB. One retrospective study demonstrated that ^18^F-FDG uptake patterns in lymph node TB were indistinguishable from those seen in lymphoma, sarcoidosis, and metastatic disease, underscoring the risk of false-positive diagnoses [46]. Consequently, improving evaluation of tuberculous lymphadenitis may require standardization and validation of multimodal imaging approaches as well as the integration of advanced analytical methods. Supporting this direction, Zhang et al. [51] developed a deep learning-based ultrasound radiomics model capable of differentiating drug-resistant from drug-sensitive lymph node TB, highlighting the potential of AI-assisted, noninvasive imaging to improve current diagnostic strategies.

## 8. Abdominal and Hepatic Tuberculosis

Abdominal TB accounts for approximately 11–16% of EPTB cases and involves the intestinal tract, peritoneum, mesentery, and other abdominal organs. The liver is the least commonly affected organ, with hepatic TB representing less than 1% of abdominal TB cases [52]. Clinical presentation is nonspecific, with symptoms including fever, chills, ascites, abdominal pain, and bowel obstruction, often obscuring diagnosis. As histopathologic confirmation is often required, imaging modalities, including CT and ultrasound, are critical for identifying abdominal TB. However, conventional imaging lacks pathognomonic features, and efforts are ongoing to identify more specific markers to improve early and accurate diagnosis [53,54,55,56].

### 8.1. Ultrasound

Ultrasound is a first-line imaging modality in the evaluation of abdominal TB due to its noninvasiveness and utility as a point-of-care tool, particularly in resource-limited settings. Despite these advantages, important limitations exist. A systematic review evaluating the diagnostic accuracy of abdominal ultrasound for abdominal TB reported only moderate sensitivity and specificity, based on very low-certainty evidence [57]. These findings indicate that ultrasound alone may not reliably confirm or exclude abdominal TB, especially in immunocompromised populations. These limitations are further highlighted through a prospective study comparing the performance of ultrasound relative to CT in evaluating patients with suspected abdominal TB. Ultrasound demonstrated lower sensitivity, specificity, and overall diagnostic accuracy compared to CT. Diagnostic performance of ultrasound and CT are summarized in Table 5. Additionally, ultrasound performance is well-documented to be limited by acoustic interference from bowel gas and focal lesion non-visualization [58]. Given these diagnostic limitations, CT plays a central role in further characterization of disease extent and diagnosis.

### 8.2. CT

**Figure 5:**
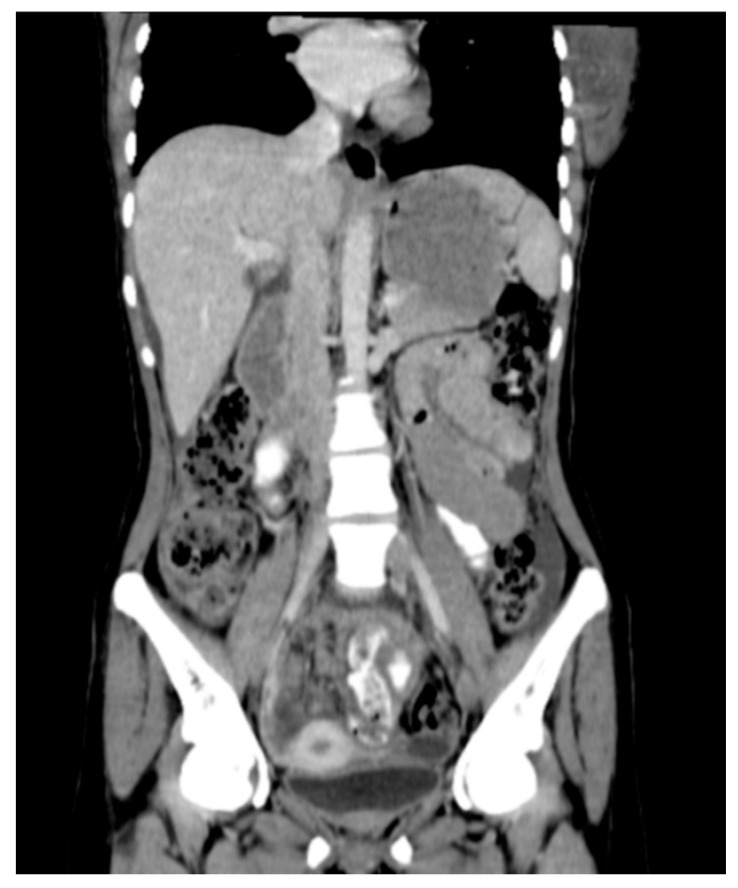
Contrast-enhanced coronal CT image of the abdomen demonstrating imaging features consistent with peritoneal TB, including peritoneal thickening and nodularity with associated ascites. These findings are characteristic of tuberculous peritonitis and may mimic peritoneal carcinomatosis. Case courtesy of Dalia Ibrahim, Radiopaedia.org, rID: 216518, licensed under CC-NC-BY-SA 3.0 [61].

Conventional CT imaging offers greater sensitivity than ultrasound in identifying and characterizing abdominal TB lesions, specifically for detecting lymphadenopathy, bowel involvement, and peritoneal disease. CT has also demonstrated improved detection of hepatic and splenic granulomas with greater frequency compared to ultrasound [58]. Furthermore, CT may reveal morphological patterns that aid in differentiating peritoneal TB from malignant mimics. For example, diffuse omental or peritoneal nodularity is observed more frequently in peritoneal TB, whereas malignant processes such as peritoneal carcinomatosis commonly display a focal-mass like or classic “omental-caking” pattern (Figure 5) [59]. Similarly, ovarian capsular change and attenuation have been reported more in peritoneal carcinomatosis secondary to ovarian cancer than in peritoneal TB [60].

Despite these advantages, distinguishing hepatic and abdominal TB from malignancy or other pathologies remains challenging. CT findings such as hypodense lesions and capsular retractions may be present in both hepatic TB and intrahepatic cholangiocarcinoma or liver metastasis [54]. Additional analyses of hepatic TB found that imaging features alone, including multiple small hypodense lesions with minimal contrast enhancement and PET uptake, frequently overlap with those of lymphoma or other liver malignancies, limiting diagnostic utility of imaging alone and necessitating histopathologic confirmation [52,55].

Diagnostic complexity is further compounded by paradoxical reactions during treatment. In a retrospective study of patients with perihepatic or hepatic TB, new or enlarging ring-enhancing lesions appeared on CT during anti-TB treatment, mimicking disease progression or treatment failure. This study highlights the value of using CT for longitudinal monitoring while underscoring its limitations in differentiating inflammatory responses from active disease [53]. Shared radiologic findings of abdominal TB mimics are summarized in Table 6.

To address these diagnostic limitations, emerging approaches such as CT texture analysis and machine learning have been explored. Radiomic analyses of omental lesions have demonstrated the ability to differentiate peritoneal TB from peritoneal carcinomatosis [56]. Similarly, a multicenter CT-based machine learning model has shown promise in distinguishing these conditions, though further validation is still required [62]. These studies illustrate a shift toward quantitative imaging and AI-assisted methods to improve diagnostic accuracy when clinical presentation and conventional imaging findings are inconclusive.

## 9. Genitourinary Tuberculosis

Genitourinary tuberculosis (GTB) manifests in the organs of the genitourinary (GU) system of both males and females, accounting for about 20% of all known EPTB cases [63]. Early diagnostic evaluation, coupled with appropriate therapeutic intervention, is crucial to minimize the risk of severe complications and associated mortality. If left untreated, GTB can potentially lead to obstructive uropathy, hydronephrosis, and renal failure [63]. GTB is challenging to diagnose due to its diverse clinical and radiological presentations, which can overlap with or be mistaken for other inflammatory or malignant disease processes. However, recent studies have shown that imaging has substantially improved diagnostic accuracy and clinical decision-making in GTB. Multimodality imaging incorporating both conventional and advanced imaging techniques, such as MRI and CT, has demonstrated sensitivities as high as 91.4% for GTB [64].

### 9.1. Computed Tomography

CT remains the primary imaging modality for evaluation and diagnosis of GTB in both males and females, mainly due to its widespread availability and ability to provide comprehensive assessment of the urinary tract and adjacent structures. For instance, CT has been utilized to detect common presentations of renal TB, including renal calcifications and uneven caliectasis resulting from fibrosis and obstruction [7]. Renal calcifications are present in almost 50% of GTB cases, and CT further enables evaluation of intra-renal and extra-renal spread of disease [7]. Ureteral TB, which usually results from secondary spread of renal TB, is also frequently diagnosed with CT. CT imaging commonly shows ureteral wall thickening with increased enhancement, accompanied by periureteral fibrosis and calcifications [7]. Collectively, these imaging features highlight the central role of CT in the diagnosis of GTB, as it reliably identifies characteristic abnormalities of GTB and delineates the extent of disease involvement.

#### 9.1.1. CT in Diagnosing Male GTB

CT has proven to be useful in diagnosing GTB in various locations of the male GU system. A clinical study by Fu et al. [65] evaluated prostate CT images from 11 male patients with disseminated pulmonary and prostatic TB, finding hypodense prostatic lesions accompanied by annular enhancement or prominent enhancement of the surrounding tissue on contrast-enhanced CT. Follow-up CT scans were just as useful in monitoring treatment response of prostate TB, revealing decreased lesion size and surrounding enhancement [65]. Furthermore, CT imaging has demonstrated diagnostic value in detecting EPTB in the seminal ducts of the male GU system. Qi et al. [66] analyzed CT images of 89 males with a history of seminal duct TB and surgical intervention and discovered three classifications of seminal duct TB: intraductal lesions, dilation of the lumen, and vas deferens wall thickening [66]. In addition to characterizing common radiologic findings, the study also assessed the consistency between CT imaging results and corresponding pathological results. The results demonstrated that CT achieved a sensitivity of 63.89% and a specificity of 80.01%, indicating moderate sensitivity and relatively high specificity for the diagnosis of seminal duct TB and its characteristic imaging features [66]. Despite the positive findings shown from both studies, it is important to recognize that seminal duct and prostatic TB are often asymptomatic and may only be detected incidentally on imaging [66]. This highlights that CT alone is insufficient as a definitive diagnostic modality, suggesting the necessity of pathologic confirmation or multimodal imaging.

#### 9.1.2. CT in Diagnosing Female GTB

GTB may also involve the female reproductive tract, where CT has demonstrated utility in diagnosing key imaging patterns. In a retrospective analysis of 26 women with fallopian tubal tuberculosis (FTTB), CT imaging revealed characteristic findings, including dilated fallopian tubes with irregular, thickened walls and occasional calcifications [67]. In many cases, these tubal abnormalities were also accompanied by ascites with high CT attenuation and linear peritoneal enhancement reflecting concomitant TB peritonitis [67]. While the combination of these features is highly suggestive of FTTB, the study was limited by a relatively low rate of histopathologic confirmation, which was achieved in less than half of cases [67]. Similar findings were reported in a study conducted by Sharma et al. [68], which assessed the role of CT imaging in the diagnosis of female genital TB with adnexal masses. Ascites along with peritoneal enhancement and thickening were observed in 42% of the 33 cases, further supporting the association between these CT features and underlying TB involvement of the female genital tract [68]. In summation, these findings suggest that recognition of specific CT patterns aids in confirming clinical suspicion for female GTB and prompts timely further diagnostic evaluation, especially in patients with nonspecific presentations.

### 9.2. Magnetic Resonance Imaging

**Figure 6:**
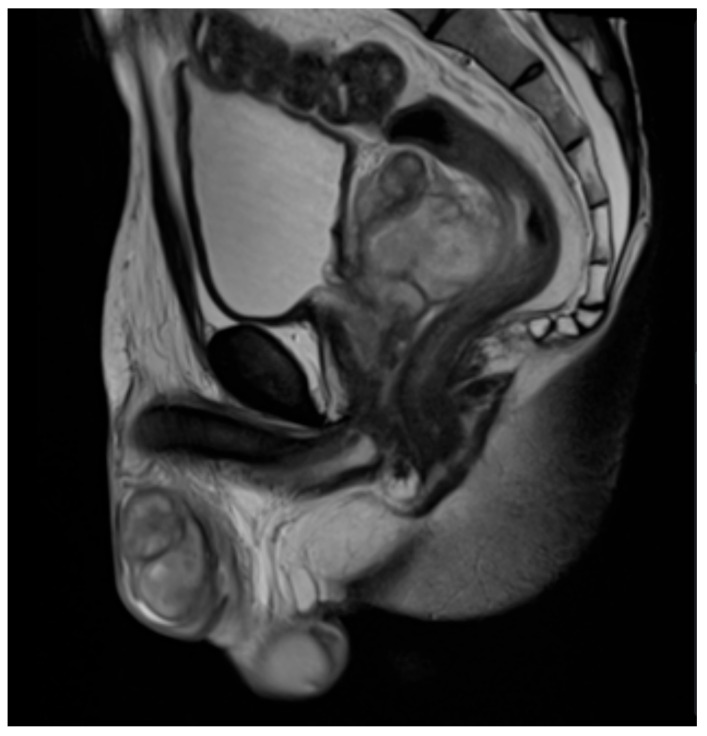
T2-weighted MRI displaying fluid collections in the right testicle, epididymis, and ductus deferens. Inflammation extends to the seminal vesicles and prostate, suggesting abscess formation characteristically seen in seminal vesicle and prostate involvement of TB. Heterogeneous contrast enhancement is also visualized. Case courtesy of Lam Van Le, Radiopaedia.org, rID: 206329, licensed under CC-NC-BY-SA 3.0 [71].

MRI serves as an important complementary imaging modality in the diagnosis of GTB in both men and women, often offering superior soft-tissue contrast for detailed assessment of disease extent. In male reproductive involvement, prostatic TB on MRI can often present as a diffuse process with glandular enlargement (diffuse form), although focal nodular involvement may also be seen (nodular form), and with both types demonstrating mild to moderate contrast enhancement (Figure 6) [69]. Disease spread to the seminal vesicles is suggested by inflammatory changes such as wall thickening or the presence of abscesses and granulomas (Figure 6) [69]. Tuberculous involvement of the epididymis is highlighted by areas of low signal intensity on T2-weighted images, which correspond with inflammatory processes like fibrosis and caseation (Figure 6) [69]. Similar to its use in male GBT, MRI plays a more selective and supportive role rather than serving as a standalone diagnostic tool in female GTB. Another study by Sharma et al. [70] evaluated the effectiveness of MRI in diagnosing tuberculous tubo-ovarian masses in female GTB across 33 patients, with findings dominated by the presence of pelvic masses in 100% of patients and right or left adnexal masses and bilateral tubo-ovarian masses in 33.33% of patients. However, these appearances are nonspecific and can potentially overlap with chronic pelvic inflammatory disease or gynecologic malignancies [70]. Thus, although MRI has high sensitivity in detecting tuberculous lesions, it offers limited independent diagnostic or prognostic value in female GTB and should be used primarily as a supplementary imaging test [70]. Radwan et al. [7] further supported the value of MRI as an adjunct modality to CT for GTB diagnosis as well, mainly because of its improved soft-tissue assessment. Overall, MRI has its greatest utility in specific cases where higher soft-tissue resolution is needed to aid in differentiating benign from malignant disease processes.

### 9.3. Contrast-Enhanced Ultrasound

Ultrasound, specifically CEUS, has gained attention as a useful adjunct in select cases of GTB, especially when standard imaging is inaccessible or when differentiating infectious lesions from malignancy is necessary [8]. Among its many benefits, CEUS provides real-time imaging without the use of ionizing radiation. In prostate TB, transrectal CEUS has been shown to detect lesions that are poorly visualized on conventional 2D ultrasound, with lesions often appearing as hypoechoic or non-enhanced areas corresponding with underlying granulomas and caseous necrosis [8]. Although CEUS findings can be variable and are not specific enough to completely replace histopathology, these findings show promise in guiding targeted biopsy and distinguishing prostatic TB from other prostatic pathologies [8]. Similarly, CEUS has also shown value in characterizing TB involvement of the vas deferens. Images typically display heterogeneous patterns or non-uniform enhancement compared to malignant inguinal lymph nodes, which often exhibit more homogeneous enhancement [72]. Key findings of GTB across different imaging modalities, including CEUS, are shown in Table 7. While CEUS is more limited in scope and operator-dependent compared with CT or MRI, these studies illustrate how advanced ultrasound techniques can provide helpful insight in resource-limited settings that require a faster, safer, and cheaper alternative in diagnostically uncertain cases.

## 10. Cutaneous and Soft Tissue Tuberculosis

Cutaneous tuberculosis (CTB), often referred to as the “Great Imitator,” is easily misdiagnosed as conditions such as sporotrichosis, sarcoidosis, or hidradenitis suppurativa [73,74,75]. Clinically, CTB most often presents as ulceration or subcutaneous nodules that can be difficult to distinguish from other diseases of the skin, which can result in delayed diagnosis and treatment [74,76]. Given that routine TB diagnostics, such as Ziehl-Nielsen staining, QuantiFERON-TB assays, and cultures, may be negative or take several weeks to yield results in patients with CTB, greater use of imaging modalities may improve diagnostic accuracy, as multimodal imaging enables noninvasive localization and assessment of the extent of the lesion, which can be particularly useful in cases where biopsy access is limited or when differentiation from neoplastic or inflammatory conditions is difficult [73,74,75,76].

### 10.1. Ultrasound

Although the role of imaging in the diagnosis of CTB remains limited and underexplored, emerging evidence suggests a potential role for ultrasound in lesion assessment. A recent 2025 study comparing 59 cases of CTB and 59 pulmonary TB cases reported subcutaneous, irregular hypoechoic regions on ultrasound, corresponding to clinically evident lesions [76].

### 10.2. Computed Tomography

**Figure 7:**
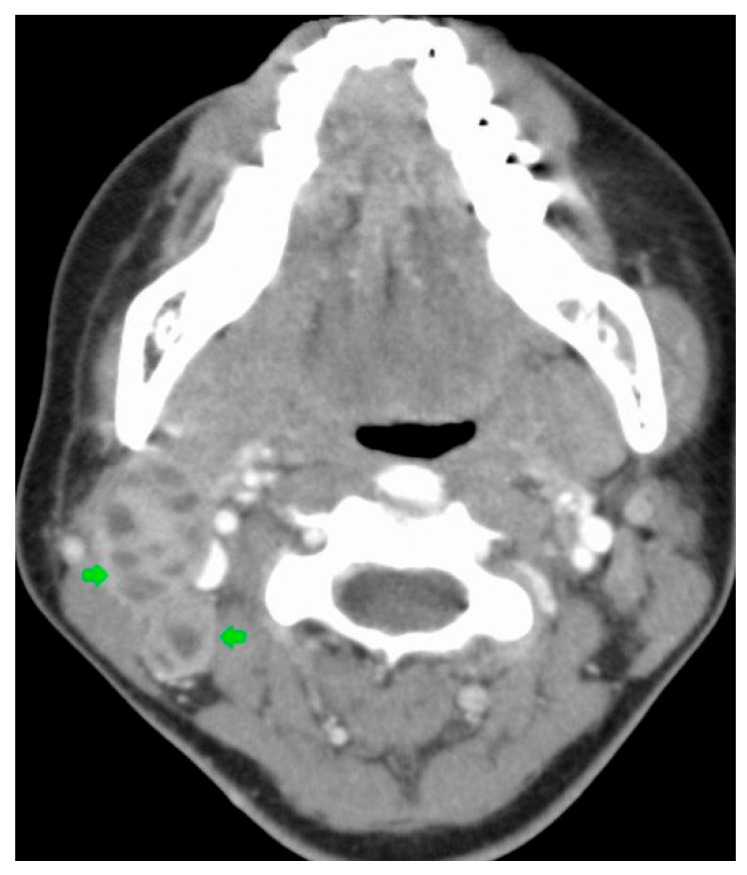
Contrast-enhanced CT of the cervical region showing enlarged lymph nodes with low-density centers (arrows), consistent with tuberculous cervical lymphadenitis. These imaging findings illustrate the underlying nodal pathology from which scrofuloderma may develop through contiguous spread to the overlying skin. Case courtesy of Frank Gaillard, Radiopaedia.org, rID: 9766, licensed under CC-NC-BY-SA 3.0 [79].

In the same cohort, CT demonstrated localized soft tissue swelling and flaky low-density shadows in patients with CTB [76]. Case reports further illustrate the utility of CT in assessing disease extent and excluding alternative diagnoses. In a 2023 case of scrofuloderma, a subtype of CTB, CT imaging excluded pulmonary involvement and identified regional lymphadenopathy, findings that supported clinical and histopathological correlation in establishing the diagnosis [77]. Similarly, in a 2022 case of scrofuloderma in a 70-year-old female patient, CT contributed to the evaluation of both soft-tissue and pulmonary involvement when used in conjunction with other imaging modalities, including CXR and MRI [73]. Because scrofuloderma typically arises from contiguous spread from an underlying infected lymph node, CT may demonstrate enlarged cervical lymph nodes with low-density centers, consistent with findings seen in tuberculous cervical lymphadenitis (Figure 7) [78,79].

### 10.3. Magnetic Resonance Imaging

MRI findings in CTB primarily relate to the assessment of deep soft tissue and osseous involvement. In the cohort study by Liu et al. [76], MRI demonstrated evidence of bone or soft tissue extension in affected patients [76]. MRI also supported diagnostic decision-making in individual cases, including a 25-year-old male with suspected scalp CTB and intracranial involvement, where imaging findings demonstrated the extent of disease and supported the initiation of empirical tuberculostatic therapy [80].

### 10.4. Radiography and Nuclear Medicine

The use of conventional radiography and nuclear medicine imaging has been described primarily in case reports of CTB, illustrating how imaging may support diagnosis and management when conventional microbiological testing is inconclusive [81,82]. For example, in a 19-year-old male with multiple scrofuloderma and tuberculosis dactylitis, plain radiography revealed osseous involvement and helped characterize the extent of disease, complementing histopathology in establishing the diagnosis [81]. Similarly, in a 69-year-old male with atypical cutaneous lesions and inconclusive histology, ^18^F-FDG PET/CT identified both cutaneous and pulmonary involvement, providing supportive evidence for the diagnosis [82]. Although nuclear medicine imaging has been reported less frequently in CTB, the use of multimodal imaging with ^18^F-FDG PET/CT may provide valuable diagnostic context for clinicians, potentially supporting more targeted and timely clinical management of CTB.

### 10.5. CTB Discussion

Overall, the examples in this section suggest that even when imaging does not directly visualize characteristic CTB lesions, it remains valuable for excluding alternative diagnoses and guiding further diagnostic work-up, particularly in the assessment of disease spread and extracutaneous involvement, although imaging findings are often nonspecific [73,76]. Key imaging findings across imaging modalities are summarized in Table 8 [73,76,77,80,81,82]. Future research, ideally through larger, systematic studies, is needed to clarify the role of imaging in enhancing diagnostic accuracy, characterizing the extent of disease, and informing clinical management of CTB.

## 11. Rare Manifestations

EPTB can involve virtually any organ system, but certain presentations are particularly rare and often pose diagnostic challenges due to their nonspecific clinical features [83]. For example, a case report described a middle-aged woman whose contrast-enhanced CT revealed esophageal wall thickening with associated mucosal ulceration. These findings aided in the detection of primary esophageal TB, which was subsequently confirmed by pathology, illustrating the role of CT in diagnosing rare EPTB manifestations [84].

Similarly, a review of eight atypical EPTB cases affecting organs such as the gallbladder, pancreas, adrenal glands, and spinal cord demonstrated that imaging modalities including CT, ultrasound, and MRI were pivotal in detecting lesions and guiding further evaluation [83]. Additionally, a recent case of EPTB affecting the spine and paraspinal soft tissues demonstrated how CT and MRI were essential for identifying destructive vertebral lesions, paraspinal abscesses, and soft tissue involvement. These imaging findings guided biopsy and timely diagnosis, highlighting the role of advanced imaging in managing this rare manifestation [85].

Collectively, these cases, which are summarized in Table 9, underscore that while EPTB can present in unexpected locations, advanced imaging has the potential to improve diagnostic accuracy and support timely evaluation in these rare manifestations.

## 12. Conclusions

This review set out to synthesize recent advances in diagnostic imaging of EPTB. Conventional imaging modalities, including ultrasound, CT, and MRI, continue to form the foundation of EPTB identification and remain essential in diagnosis [3]. Their widespread availability and established diagnostic value allow for assessment of EPTB across organ systems. Ultrasound was found to be useful in the evaluation of lymphatic TB, with TTE serving as a useful ultrasound-based modality for detecting cardiac involvement [42,45]. CT plays a key role in the assessment of abdominal TB and GTB, whereas MRI is the preferred modality for CNS TB and tuberculous spondylitis [3,7,36,58].

Emerging imaging approaches like PET/CT, CEUS, and advanced MRI modalities, including DTI, offer promising opportunities to enhance imaging [8,16,30,49]. Although early studies demonstrate encouraging results, clinical implementation remains limited by heterogeneous study designs, lack of standardization, and restricted access in high-burden, resource-limited settings [19,39]. Despite increasing capabilities of emerging techniques, many remain cost-prohibitive for widespread use, particularly in resource-constrained settings where the TB burden is greatest. Imaging strategies should be guided by local infrastructure, population factors and cost-effectiveness. While advanced imaging is often favored in resource-rich settings, more affordable modalities paired with confirmatory testing should be explored as practical alternatives in high-burden settings [86].

Importantly, delayed diagnosis of EPTB is not solely attributable to imaging limitations but also to the variability in clinical suspicion across medical specialties. A retrospective case series by Lee et al., [87] found that patients receiving immunosuppressive therapies were more likely to be diagnosed early, while patients evaluated in surgical or gastroenterology settings experienced significant diagnostic delays, likely due to lower initial suspicion [87]. These findings highlight the need for increased education and awareness of EPTB among non-pulmonary specialties.

Imaging plays a key role in the diagnosis and management of EPTB by defining the extent of disease, supporting lesion characterization, guiding tissue sampling and enabling treatment monitoring. While imaging findings are not diagnostic in isolation and must be interpreted alongside clinical presentation and epidemiologic risk, the continued integration of organ-specific imaging strategies with emerging modalities and improved cross-specialty awareness will be essential to reduce diagnostic delays and improve outcomes in patients with EPTB.

This review has several limitations. As a narrative review, it is subject to potential selection and publication bias despite a structured literature search. The included studies were heterogeneous in design, imaging protocols, and patient populations. Additionally, many were retrospective, single-center, or limited by small sample sizes, all of which restrict the generalizability of the findings. For certain forms of EPTB, specifically cutaneous TB and other rare manifestations, the available evidence was largely limited to case reports and small case series.

Future research should focus on large, prospective studies with standardized imaging protocols to better define the diagnostic and prognostic value of imaging modalities in EPTB. Advanced imaging methods, such as ^18^F-FDG PET/CT and DTI, should continue to be explored. AI-based imaging analysis offers additional promise in improving diagnostic accuracy, but requires further validation across diverse clinical settings and clarification of its role in guiding treatment decisions and personalizing therapy for EPTB.

## Figures and Tables

**Table 1:** Summary of key findings across different imaging modalities in the diagnosis of CNS TB subtypes.

Imaging Modality	CNS TB Type	Key Findings	Source
MRI	Cranial Meningitis TB	Tuberculoma	[5]
		Meningeal enhancement	[5,23,26]
		Absence of PPBS	[24]
	Parenchymal TB	Fibrosis and sclerosis	[23]
	Spinal Meningitis TB	Linear dural enhancements	[25]
	Spinal Parenchymal TB	Linear dural enhancements, CSF loculations, arachnoiditis, tuberculoma	[29]
DTI	Cranial Meningitis TB	Reduction in FA values	[30]
		White matter changes in corpus callosum and corona radiata	[6]

**Table 2:** Summary of key findings of tuberculous spondylitis.

Imaging Modality	Key Findings	Source
MRI	Paravertebral abscesses with clear boundaries	[36]
	Enhancement of anterior longitudinal ligament	[36]

**Table 3:** Summary of key findings across different imaging modalities in the diagnosis of tuberculous pericarditis.

Imaging Modality	Key Findings	Source
TTE	Pericardial effusion	[42]
CT	Pericardial thickening, calcifications, mediastinal lymphadenopathy	[43]
^18^F-FDG PET	Pericardial uptake of ^18^F-FDG	[43]
MRI	Constrictive pericarditis	[40]

**Table 4:** Summary of key findings across different imaging modalities in the diagnosis of tuberculous lymphadenitis.

Imaging Modality	Key Findings	Source
Ultrasound	Unclear margin	[45]
	Intranodal pus	[47]
CT	Paraaortic dissemination, diameter of abdominal tuberculous lymphadenopathy	[9]

**Table 5:** Diagnostic performance of ultrasound and CT for diagnosis of abdominal TB.

Imaging Modality	Sensitivity	Specificity	Source
US	63–71%	68–78%	[57,58]
CT	92%	85%	[58]

**Table 6:** Diagnostic mimics of abdominal TB on CT imaging.

Imaging Modality	Mimic	Shared Radiologic Finding	Source
CT	Intrahepatic cholangiocarcinoma or liver metastasis	Hypodense lesions, capsular retractions	[54]
	Lymphoma	Multiple small hypodense lesions with increased PET uptake	[55]
	TB treatment failure	Ring-enhancing lesions	[53]

**Table 7:** Summary of key findings across different imaging modalities in the diagnosis of GTB subtypes.

Imaging Modality	GTB Type	Key Findings	Source
CT	Renal TB	Renal calcifications, uneven caliectasis	[7]
	Ureteral TB	Ureteral wall thickening with increased enhancement	[7]
	Prostatic TB	Hypodense prostatic lesions with annular enhancement	[65]
	Fallopian Tubal TB	Irregular thickened walls with accompanying ascites and peritoneal enhancement	[67,68]
MRI	Prostatic TB	Glandular enlargement or focal nodular involvement	[69]
	Seminal Vesicle TB	Wall thickening or presence of abscesses and granulomas	[69]
CEUS	Prostatic TB	Hypoechoic or non-enhanced areas	[8]
	Vas deferens TB	Heterogenous patterns or non-uniform enhancement	[72]

**Table 8:** Summary of key imaging findings in CTB across different modalities. CT, CXR, X-Ray, MRI, and Ultrasound can reveal characteristic features of CTB, guide biopsy, assess disease extent, and aid in differential diagnosis.

Imaging Modality	Key Findings	Source
CT	Supraclavicular soft-tissue mass infiltrating adjacent tissues consistent with scrofuloderma	[73]
	Localized soft tissue swelling; patchy low-density shadows	[76]
	Regional lymphadenopathy; excluded pulmonary involvement	[77]
CXR	Unilateral residual hilar calcification	[73]
X-ray	Blastic lesion with cortical erosions in metacarpal; lytic lesions in calcaneus and cuboid; soft tissue swelling	[81]
MRI	Extensive skin and soft tissue lesions from the neck to the apex of the lung	[73]
	Evidence of bone or soft tissue extension	[76]
	Contiguous scalp soft tissue invasion into meninges; bony skull involvement	[80]
Ultrasound	Subcutaneous irregular hypoechoic regions	[76]

**Table 9:** Summary of key imaging findings in Rare Manifestations of EPTB across different modalities.

Type of EPTB	Imaging Modality	Key Findings	Source
Esophageal	CT	Esophageal wall thickening and associated mucosal ulceration	[84]
Spine and Paraspinal Soft Tissues	CT & MRI	Destructive vertebral lesions, paraspinal abscesses, soft tissue involvement	[85]

## Data Availability

No new data were created or analyzed in this study.

## Abbreviations

AI	Artificial Intelligence
CEUS	Contrast-Enhanced Ultrasound
CNS	Central Nervous System
CSF	Cerebrospinal Fluid
CT	Computed Tomography
CTB	Cutaneous Tuberculosis
CXR	Chest X-ray
DTI	Diffusion Tensor Imaging
EPTB	Extrapulmonary Tuberculosis
eFASH	Extended Focused Assessment with Sonography for HIV and Tuberculosis
FASH	Focused Assessment with Sonography for HIV and Tuberculosis
^18^F-FDG	^18^F-fluorodeoxyglucose
FTTB	Fallopian Tubal Tuberculosis
GTB	Genitourinary Tuberculosis
GU	Genitourinary
LETM	Longitudinal Extensive Transverse Myelitis
MRI	Magnetic Resonance Imaging
TB	Tuberculosis
TTE	Transthoracic Echocardiography
PET	Positron Emission Tomography
PET/CT	Positron Emission Tomography/Computed Tomography
POCUS	Point of Care Ultrasound
PPBS	Posterior Pituitary Bright Spot
